# Long-term efficacy of the *Astragalus-Salvia miltiorrhiza* botanical drug pair in stable coronary artery disease and a nomogram for predicting frequent hospitalization: a real-world retrospective cohort study

**DOI:** 10.3389/fphar.2026.1898253

**Published:** 2026-09-04

**Authors:** Liwen Fu, Jiaxiu Han, Zu Gao, Huiyan Long, Yanzhu Liu, Yunlun Li

**Affiliations:** 1 Innovative Institute of Chinese Medicine and Pharmacy, Shandong University of Traditional Chinese Medicine, Jinan, China; 2 Rehabilitation Medical College, Shandong University of Traditional Chinese Medicine, Jinan, China; 3 The First Clinical Medical College of Shandong University of Traditional Chinese Medicine, Jinan, China; 4 Xinyang Clinic, Hangzhou, Zhejiang Province, China; 5 Acupuncture & Wellness, New York, United States

**Keywords:** Astragalus-Salvia miltiorrhiza botanical drug pair, inverse probability of treatment weighting, nomogram, real-world study, retrospective cohort study, stable coronary artery disease

## Abstract

**Objective:**

To evaluate the long-term efficacy and safety of the *Astragalus-Salvia miltiorrhiza* (ASM) botanical drug pair as an adjunctive therapy for stable coronary artery disease (SCAD) and to develop a prognostic nomogram for frequent readmissions.

**Methods:**

This real-world retrospective cohort study included 686 SCAD patients (343 receiving ASM plus standard care and 343 receiving standard care alone). Inverse probability of treatment weighting (IPTW) was applied to balance covariates. After a 5-year follow-up, biochemical parameters, primarily low-density lipoprotein cholesterol (LDL-C), and safety profiles were compared. Furthermore, multivariable logistic regression was used to construct a nomogram predicting frequent readmissions (≥3 times), and symptom networks were analyzed via the Apriori algorithm to evaluate symptom alleviation.

**Results:**

At the 5-year follow-up, compared to controls, the ASM group exhibited significantly reduced LDL-C, total cholesterol (TC), triglycerides (TG), heart rate, and monocytes, alongside elevated high-density lipoprotein cholesterol (HDL-C), free triiodothyronine (FT3), and free thyroxine (FT4) (all *P* < 0.05). Hepatic and renal functions remained unaffected (*P* > 0.05). Notably, stroke history emerged as the primary independent risk factor (*P* < 0.001) for frequent readmissions. The nomogram integrating stroke history, age, diastolic blood pressure, FT3, and hemoglobin demonstrated excellent calibration. Symptom network analysis identified “chest tightness” as the core symptom alleviated by ASM.

**Conclusion:**

ASM adjunctive therapy safely improves lipid profiles, stabilizes heart rate, and alleviates chest tightness in SCAD. The proposed nomogram offers a reliable tool for risk stratification, providing real-world evidence to optimize the integrative management of SCAD.

**Clinical Trial Registration:**

https://itmctr.ccebtcm.org.cn/mgt/dashboard, identifier TMCTR2025000058.

## 1 Introduction

Stable coronary artery disease (SCAD) is a leading cause of global mortality (GBD 2019 Diseases and Injuries Collaborators, 2020). Despite standard pharmacological therapies (e.g., antiplatelet and lipid-lowering agents), a substantial proportion of patients retain high residual cardiovascular risk (Vuorio et al., 2026). This residual risk frequently causes recurrent hospitalizations, diminishing quality of life and burdening healthcare systems. Therefore, identifying effective adjunctive therapies to optimize patient management and reduce readmission rates is clinically imperative.

Throughout the pathological progression of SCAD, dyslipidemia, particularly the abnormal elevation of low-density lipoprotein cholesterol (LDL-C), acts as the core initiating factor. It drives vascular endothelial injury, triggers localized inflammatory responses, and precipitates the instability of atherosclerotic plaques (Zhu et al., 2023). Consequently, the strict control and targeted reduction of LDL-C levels are imperative for stabilizing plaques, minimizing ischemic events, and ultimately improving clinical prognosis.

In Traditional Chinese Medicine (TCM), SCAD pathogenesis is characterized by “Qi deficiency and blood stasis” (Xin and Li, 2025). Specifically, Qi deficiency results in an inability to propel blood circulation, while blood stagnation breeds turbid stasis. The intertwining of phlegm and stasis within the collaterals ultimately obstructs the heart vessels. In modern pathological terms, this concept mirrors the SCAD progression cascade: compromised cardiac hemodynamics (“Qi deficiency”) exacerbates endothelial injury, lipid accumulation, and inflammation, ultimately driving plaque instability and vascular occlusion (“blood stasis”) (Wang and Wang, 2025). Thus, there is a plausible link between the traditional treatment of these symptoms and modern clinical interventions targeting atherosclerosis. The *Astragalus-Salvia miltiorrhiza* (ASM) botanical drug pair is a classic TCM combination utilized to “tonify Qi and activate blood circulation” in cardiovascular diseases (Yu et al., 2025). *Astragalus membranaceus* (Huang qi) promotes hemodynamics, while *Salvia miltiorrhiza* (Dan shen) improves microcirculation and alleviates ischemia. Modern pharmacological evidence suggests that ASM metabolites possess pleiotropic activities, including lipid regulation, endothelial protection, and anti-inflammatory effects (Ye, 2021). However, robust, large-scale real-world studies evaluating its long-term efficacy and prognostic impact in SCAD patients are lacking.

Therefore, we conducted a real-world retrospective cohort study to evaluate the long-term efficacy and safety of ASM in SCAD, and to develop a nomogram for predicting frequent hospitalization. To ensure scientific rigor, treatment outcomes were assessed using quantifiable clinical endpoints, primarily tracking the reduction in core lipid biomarkers (e.g., LDL-C) and the incidence of hospital readmissions. This study aims to provide robust, evidence-based support for the integrative management of SCAD and establish a clinical foundation for future mechanistic investigations into ASM.

## 2 Clinical data

### 2.1 General information

This retrospective, single-center cohort study analyzed clinical data from patients with stable coronary artery disease (SCAD) treated at the Affiliated Hospital of Shandong University of Traditional Chinese Medicine between January 2014 and January 2024. Patients were divided into two cohorts: an ASM group (receiving the Astragalus-Salvia miltiorrhiza botanical drug pair for ≥4 weeks alongside standard care) and a control group (receiving standard care alone). To minimize selection bias and confounding factors, inverse probability of treatment weighting (IPTW) was applied to balance baseline covariates between the two groups.

This study was registered with the International Traditional Medicine Clinical Trial Registry (Registration No.: ITMCTR2025000058). Furthermore, the study protocol was reviewed and approved by the Ethics Committee of the Affiliated Hospital of Shandong University of TCM [Ethics Approval No (2024) Lun Shen Di (172)-KY].

### 2.2 Botanical drug composition and quality control

In routine clinical practice, the ASM botanical drug pair was administered in various formulations (e.g., decoctions, granules, pills, and commercial preparations), maintaining a fundamental mass ratio of Astragalus membranaceus to Salvia miltiorrhiza at 2:1.

Due to the 10-year retrospective design, real-time chemical profiling (e.g., HPLC/MS) of historical batches was unfeasible. To ensure longitudinal chemical consistency and strictly adhere to the ConPhyMP statement, quality control relied on national regulatory frameworks. All botanical products were exclusively sourced from Good Manufacturing Practice (GMP)-certified enterprises regulated by the National Medical Products Administration (NMPA) of China.

Crucially, all dispensations strictly met the Chinese Pharmacopoeia (ChP) standards. Per the ChP, Astragalus is standardized by astragaloside IV (≥0.08%) and calycosin-7-O-β-D-glucoside (≥0.020%), while Salvia miltiorrhiza is standardized by salvianolic acid B (≥3.0%) and total tanshinones (tanshinone IIA, cryptotanshinone, and tanshinone I; ≥0.25%). Detailed taxonomic validation complying with ConPhyMP guidelines is summarized in Table 1.

**TABLE 1:** Validated botanical nomenclature and pharmacopoeial quality control standards of the studied botanical drugs.

Validated botanical nomenclature [family; pharmacopoeial drug name]	Part used	Major marker metabolites (limit)
*Astragalus membranaceus* (Fisch.) Bunge var. *mongholicus* (Bunge) Hsiao or *Astragalus membranaceus* (Fisch.) Bunge [Fabaceae; Astragali Radix]	Root	Astragaloside IV (≥0.080%); Calycosin-7-O-β-D-glucoside (≥0.020%)
*Salvia miltiorrhiza* Bunge [Lamiaceae; Salviae Miltiorrhizae Radix et Rhizoma]	Root and rhizome	Total of Tanshinone IIA, Cryptotanshinone, and Tanshinone I (≥0.25%); Salvianolic acid B (≥3.0%)

Botanical names and family assignments were validated against Plants of the World Online (POWO, http://www.plantsoftheworldonline.org; accessed on 8 August 2026). Pharmacopoeial drug names, medicinal parts, and marker compound limits were based on the ChP.

### 2.3 Diagnostic criteria

The diagnosis of SCAD followed the 2018 guidelines of the Chinese Medical Association (Shen et al., 2019). Confirmation required coronary angiography (CAG) or computed tomography angiography (CCTA) demonstrating ≥50% luminal stenosis in at least one major coronary artery (left main, left anterior descending, left circumflex, or right coronary artery), alongside stable angina or asymptomatic myocardial ischemia.

The TCM diagnosis of “Qi deficiency and blood stasis” syndrome was identified according to the 2019 guidelines of the China Association of Chinese Medicine (Guidelines for the diagnosis and treatment of stable angina pectoris of coronary heart disease in The Cardiovascular Branch of the China Association of Chinese Medicine, 2019). Primary symptoms included chest pain, tightness, or discomfort, while secondary symptoms comprised palpitations, shortness of breath, fatigue, and exertional sweating. Characteristic signs presented as a dark purple tongue (with or without petechiae/ecchymosis) and a thready, choppy pulse.

### 2.4 Inclusion criteria

1. Met the diagnostic criteria for SCAD, and it was recorded as the primary diagnosis;
2. Met the TCM diagnostic criteria for chest bi (chest obstruction) with Qi deficiency and blood stasis syndrome;
3. Aged ≥18 years;
4. Complete electronic medical record (EMR) data.

### 2.5 Exclusion criteria

1. Admission diagnosis complicated by acute myocardial infarction (AMI) or unstable angina requiring emergency revascularization;
2. Pregnant or lactating women;
3. Concurrent severe hepatic or renal dysfunction, malignant tumors, or severe immune system diseases.

## 3 Methods

### 3.1 Sample size calculation

An *a priori* sample size estimation was performed to ensure adequate statistical power for comparing clinical efficacy and hypothesis testing of core continuous physiochemical parameters between the two groups. Based on previous studies, the overall effective rate for SCAD is approximately 75% with conventional therapy alone, compared to 84% when combined with Chinese medicine. The sample size was calculated using the following formula:

$$n=\frac{{\left[Z_{\alpha }\sqrt{2\bar{p}\left(1‐\bar{p}\right)}+Z_{\beta }\sqrt{p_{1}\left(1‐p_{1}\right)+p_{0}\left(1‐p_{0}\right)}\right]}^{2}}{{\left(p_{1}‐p_{0}\right)}^{2}}$$

where $p_{0}$ represents the effective rate of the control group (set at 75%); $p_{1}$ represents the effective rate of the exposure group (set at 84%); and $\bar{p}$ is the pooled average of the two effective rates. $Z_{\alpha }$ and $Z_{\beta }$ correspond to the quantiles of the standard normal distribution for α and β , respectively. Based on a one-sided test with α=0.05 and β=0.1 , the critical values were set to =1.65 and =1.28 . The calculation indicated that a minimum standard sample size of 343 patients was required for both the exposure and control groups.

### 3.2 Index date and observation window

The date of initial hospital admission for SCAD was defined as the index date (cohort entry), from which all baseline clinical data were extracted. To ensure standardized evaluation, a fixed 5-year observation window was established for all patients. Follow-up commenced on the index date and concluded at the 5-year mark, the time of death, or the last recorded medical encounter, whichever occurred first.

### 3.3 Outcome measures

#### 3.3.1 Baseline characteristics

Baseline variables were assessed to ensure comparability between the two groups, including age, gender, employment status, smoking and alcohol history, comorbidities (hypertension, diabetes mellitus, stroke), heart rate, blood pressure, lipid profiles, liver, renal, and thyroid functions, and complete blood count (CBC).

#### 3.3.2 Clinical outcomes

1. Primary outcome: The primary endpoint was the change in LDL-C levels from baseline to the end of the observation window (the 5-year mark or the last valid laboratory record).
2. Secondary outcomes: Secondary endpoints included changes in heart rate, blood pressure, other lipid parameters, liver, renal, and thyroid functions, and CBC from baseline to the end of the observation window.

#### 3.3.3 Safety assessment

Safety was evaluated using the laboratory parameters designated as secondary outcomes. Potential systemic toxicity and bleeding risks were assessed through the continuous monitoring of liver function (specifically ALT and AST), renal function (Scr and BUN), and complete blood count (CBC) throughout the observation window.

### 3.4 Univariate analysis for frequent hospitalization

Frequent hospitalization (defined as ≥3 admissions) was designated as a key prognostic endpoint. To identify potential risk factors, baseline variables were compared between the frequent and non-frequent hospitalization groups. Continuous variables were analyzed using Student’s *t*-test, and categorical variables were evaluated using the Chi-square test.

### 3.5 Multivariable logistic regression and model construction

Variables demonstrating statistical significance (*P* < 0.05) in the univariate analysis were incorporated into a multivariable logistic regression model to determine independent predictors of frequent hospitalization. Results are presented as odds ratios (ORs) with 95% confidence intervals (CIs). Finally, a clinical nomogram was constructed using the identified independent predictors to facilitate individualized risk estimation.

### 3.6 Model performance evaluation and internal validation

The performance of the constructed nomogram was evaluated for discrimination and calibration. Discrimination was quantified using the area under the receiver operating characteristic curve (AUC). Calibration was visually assessed via calibration curves and internally validated using a bootstrap method with 1,000 resamples to correct for overfitting. Additionally, the Hosmer-Lemeshow goodness-of-fit test was applied to quantitatively evaluate model calibration.

### 3.7 Symptom co-occurrence network analysis

To explore the intrinsic relationships among clinical symptoms in patients with SCAD, association rule analysis was conducted using the Apriori algorithm. Minimum thresholds for support and confidence were set at 15% and 70%, respectively. Based on occurrence frequencies, symptoms were categorized into core, bridging, and peripheral nodes to delineate their topological network patterns. Data processing and network visualization were executed using R and Cytoscape software.

### 3.8 Statistical analysis

Statistical analyses were performed using SPSS version 27.0 and R software version 4.4.3. Continuous variables were presented as means ± standard deviations (SDs) or medians with interquartile ranges (IQRs) depending on their distribution. Inter-group comparisons were conducted using the Student’s t-test (independent or paired) for normally distributed data, and appropriate non-parametric tests (e.g., Mann-Whitney U or Wilcoxon signed-rank test) for non-normally distributed and discrete count variables. Categorical variables were expressed as frequencies with percentages and compared via the Chi-square test.

As previously described, multivariable logistic regression and subsequent nomogram construction were executed using the rms package in R. Model discrimination was quantified via the concordance index (C-index), alongside calibration curves. All statistical tests were two-sided, and a *P*-value <0.05 was considered statistically significant.

## 4 Results

### 4.1 Baseline characteristics

The final matched cohort comprised 686 patients, evenly distributed between the exposure and control groups (n = 343 each). Following the matching process, there were no statistically significant differences between the two groups regarding demographics, lifestyle factors, or comorbidities (including hypertension, diabetes, and stroke). Furthermore, baseline vital signs and comprehensive laboratory parameters—including lipid, hepatic, renal, and thyroid profiles, as well as routine blood tests—were highly comparable between the cohorts (all *P* > 0.05). Overall, the baseline characteristics of the study population were well-balanced (Table 2).

**TABLE 2:** Baseline characteristics of the matched study population.

Variables		Control group (n = 343)	Exposure group (n = 343)	*P* value
Age (years)		69.4 ± 10.6	69.8 ± 9.7	0.603
Sex n (%)	Male	131 (38.2)	141 (41.1)	0.482
	Female	212 (61.8)	202 (58.9)	0.482
Hypertension, n (%)		223 (65.0)	244 (71.1)	0.101
Diabetes mellitus, n (%)		106 (30.9)	102 (29.7)	0.726
Stroke, n (%)		106 (30.9)	114 (33.2)	0.585
Employment status n (%)	Retired	284 (82.8)	305 (88.9)	0.151
	Employed	32 (9.3)	18 (5.2)	0.151
Smoking history, n (%)		56 (16.3)	56 (16.3)	1.000
Alcohol drinking, n (%)		37 (10.8)	45 (13.1)	0.410
Heart rate (bpm)		72 (66,80)	72 (66,78)	0.139
Lipid profile	TC (mmol/L)	4.34 (3.5,5.16)	4.14 (3.40,5.16)	0.495
	TG (mmol/L)	1.27 (0.93,1.69)	1.35 (1,1.84)	0.099
	LDL-C (mmol/L)	2.42 (1.78,3.09)	2.44 (1.85,3.19)	0.353
	HDL-C (mmol/L)	1.12 (0.93,1.33)	1.08 (0.94,1.25)	0.076
Blood pressure	DBP mmHg)	77 (70,84)	77 (69,84)	0.901
	SBP(mmHg)	132 (122,144)	133 (123,144)	0.347
Liver function	ALT (U/L)	16 (13,25)	17 (13,24)	0.448
	AST (U/L)	19 (16,24)	19 (16,24)	0.974
Renal function	SCr(umol/L)	62 (54,74)	64 (55,77)	0.156
	BUN(mmol/L)	4.99 (4.27,6.05)	5.01 (4.24,6)	0.854
	UA (μmol/L)	297 (243,355)	296 (244,361)	0.919
	eGFR (mL/min/1.73m^2^)	88.99 (79.22,97.57)	87.82 (78.17,96.12)	0.205
Thyroid function	TSH(mlU/L)	2.10 (1.39,3.06)	2.13 (1.51,3.15)	0.268
	FT3 (pmol/L)	4.22 (3.65,4.65)	4.11 (3.63,4.56)	0.112
	FT4 (pmol/L)	15.50 (14.10,17.20)	15.62 (14.10,17.65)	0.650
Routine blood test	Absolute monocyte count (×10^9^/L)	0.47 (0.38,0.55)	0.46 (0.37,0.54)	0.087
	Monocyte percentage (%)	7.5 (6.37,8.8)	7.60 (6.37,8.50)	0.624
	Platelet count (×10^9^/L)	213 (181,249)	212 (177,248)	0.861
	Hemoglobin (g/L)	133 (124,145)	132 (123,142)	0.094

Data are presented as mean ± standard deviation (SD) for continuous variables with normal distribution, median (interquartile range, IQR) for continuous variables with skewed distribution, or number (percentage) for categorical variables.

Abbreviations: SBP, systolic blood pressure; DBP, diastolic blood pressure; TC, total cholesterol; TG, triglycerides; LDL-C, low-density lipoprotein cholesterol; HDL-C, high-density lipoprotein cholesterol; ALT, alanine aminotransferase; AST, aspartate aminotransferase; SCr, serum creatinine; BUN, blood urea nitrogen; UA, uric acid; eGFR, estimated glomerular filtration rate; TSH, thyroid-stimulating hormone; FT3, free triiodothyronine; FT4, free thyroxine.

### 4.2 LDL-C levels at the end of follow-up

At the end of the follow-up period, patients in the exposure group exhibited significantly lower median LDL-C levels compared to those in the control group (1.97 [IQR: 1.49–2.48] vs. 2.17 [IQR: 1.63–2.88] mmol/L, *P* = 0.003).

### 4.3 Other clinical parameters at follow-up

Compared to the control group, the exposure group demonstrated significantly higher levels of high-density lipoprotein cholesterol (HDL-C), free triiodothyronine (FT3), and free thyroxine (FT4). Concurrently, significant reductions were observed in total cholesterol (TC), triglycerides (TG), heart rate, absolute monocyte count, and monocyte percentage within the exposure group (all *P* < 0.05).

Conversely, no significant inter-group differences were detected regarding blood pressure (systolic and diastolic), hepatic and renal function markers (ALT, AST, Scr, BUN, uric acid, eGFR), TSH, or other hematological indices (platelet count and hemoglobin) (all *P* > 0.05) (Table 3).

**TABLE 3:** Comparison of clinical indicators between the two groups at the end of follow-up.

Variables		Control group (n = 343)	Exposure group (n = 343)	*P* value
Lipid profile	HDL-C (mmol/L)	1.09 (0.96,1.28)	1.15 (1.04,1.32)	<0.001
	TC (mmol/L)	3.9 (3.29,4.92)	3.35 (2.85,3.89)	<0.001
	TG (mmol/L)	1.21 (0.94,1.66)	1.17 (0.84,1.51)	0.038
Heart rate (bpm)		72 (66,79)	70 (64,75)	<0.001
Blood pressure	DBP(mmHg)	76 (69,84)	76 (70,85)	0.915
	SBP(mmHg)	134 (121,146)	132 (122,147)	0.769
Liver function	ALT (u/L)	17 (13,23)	16 (12,24)	0.195
	AST (u/L)	20 (16,24)	20 (17,24)	0.317
Renal function	SCr(umol/L)	58 (49,70)	60 (50,72)	0.156
	BUN(mmol/L)	4.95 (4.12,6.10)	4.92 (4.07,5.96)	0.557
	UA (umol/L)	290 (241,347)	289 (237,368)	0.570
	eGFR (mL/min/1.73m^2^)	91.64 (84.42,100.27)	91.25 (82.80,99.28)	0.286
Thyroid function	TSH(mlU/L)	2.14 (1.38,2.84)	2.14 (1.38,2.84)	0.412
	FT3 (pmol/L)	4.12 (3.61,4.51)	4.17 (3.72,4.58)	<0.001
	FT4 (pmol/L)	15.34 (13.90,17.22)	16 (14.40,17.79)	0.003
Routine blood test	Absolute monocyte count (×10^9^/L)	0.47 (0.38,0.58)	0.45 (0.37,0.55)	0.045
	Monocyte percentage (%)	8.1 (6.8,9.8)	7.8 (6.7,8.9)	<0.001
	Platelet count (×10^^9^/L)	207 (171,243)	205 (167,241)	0.345
	Hemoglobin (g/L)	130 (119,143)	131 (120,142)	0.283

Data are presented as median (interquartile range, IQR).

Abbreviations: TC, total cholesterol; TG, triglycerides; HDL-C, high-density lipoprotein cholesterol; SBP, systolic blood pressure; DBP, diastolic blood pressure; ALT, alanine aminotransferase; AST, aspartate aminotransferase; SCr, serum creatinine; BUN, blood urea nitrogen; UA, uric acid; eGFR, estimated glomerular filtration rate; TSH, thyroid-stimulating hormone; FT3, free triiodothyronine; FT4, free thyroxine.

### 4.4 Objective safety evaluation

Throughout the observation period, continuous monitoring of laboratory parameters revealed no abnormal trends or clinically significant deviations indicative of systemic toxicity or increased bleeding risk. Specifically, indices of hepatic function (e.g., ALT, AST) and renal function (e.g., Scr, BUN), along with routine hematological profiles, exhibited no clinically meaningful deterioration from baseline to the end of follow-up (Table 3). Collectively, these objective findings underscore the favorable safety and tolerability profile of the treatment.

### 4.5 Baseline characteristics and univariate analysis of frequently hospitalized

Although the ASM botanical drug pair demonstrated multidimensional physiological benefits—particularly in optimizing lipid profiles and stabilizing thyroid and cardiac parameters—frequent hospitalization remains a critical challenge that severely compromises the long-term prognosis of patients with stable coronary artery disease (SCAD). To bridge these physiological improvements with long-term clinical outcomes, we systematically screened for baseline risk factors associated with frequent hospitalization (defined as ≥ 3 admissions).

Univariate analysis was initially performed to identify baseline variables correlated with frequent hospitalization. The results revealed significant differences between the frequent and non-frequent hospitalization cohorts across five variables: age, diastolic blood pressure (DBP), FT3, hemoglobin, and a history of stroke. Specifically, patients in the frequent hospitalization group were significantly older (P < 0.001) and exhibited a markedly higher prevalence of prior stroke (P < 0.001). Conversely, baseline levels of DBP, FT3, and hemoglobin were significantly lower in these frequently hospitalized patients (all *P* < 0.05). No statistically significant differences were observed for the remaining variables between the two groups (Table 4).

**TABLE 4:** Univariate analysis of baseline variables associated with frequent hospitalization in patients with SCAD.

Variables		Infrequent hospitalization group	Frequent hospitalization group	*P* value
Age (years)		68.4 ± 10.4	71.1 ± 9.8	<0.001
Blood pressure	SBPmmHg	137.6 ± 54.1	133.3 ± 17.0	0.149
	DBPmmHg	78.1 ± 10.9	76.2 ± 11.4	0.029
Heart rate (bpm)		72.9 ± 11.0	73.1 ± 11.0	0.822
Lipid profile	TC (mmol/L)	4.3 ± 1.2	4.4 ± 1.3	0.756
	TG (mmol/L)	1.5 ± 0.7	1.5 ± 1.1	0.945
	LDL-C (mmol/L)	2.5 ± 0.9	2.5 ± 1.0	0.779
	HDL-C (mmol/L)	1.1 ± 0.3	1.1 ± 0.3	0.103
Liver function	ALT (u/L)	20.0 ± 11.6	19.8 ± 10.8	0.813
	AST (u/L)	20.8 ± 7.7	21.3 ± 8.8	0.464
Renal function	SCr(umol/L)	65.7 ± 15.2	65.1 ± 15.5	0.587
	eGFR (mL/min/1.73m^2^)	89.2 ± 15.3	88.5 ± 14.5	0.556
	BUN(mmol/L)	5.04 (4.28,6.04)	4.92 (4.24,5.99)	0.551
	UA (umol/L)	310.0 ± 86.1	304.7 ± 88.0	0.424
Thyroid function	FT3 (pmol/L)	4.20 (3.69,4.63)	4.04 (3.58,4.56)	0.023
	FT4 (pmol/L)	15.47 (13.90,17.16)	15.70 (14.35,17.50)	0.103
	TSH(mIU/L)	2.10 (1.47,3.07)	2.16 (1.43,3.06)	0.923
Routine blood test	Absolute monocyte count (×10^9^/L)	0.5 ± 0.1	0.5 ± 0.1	0.260
	Monocyte percentage (%)	7.4 ± 1.6	7.6 ± 1.5	0.161
	Hemoglobin (g/L)	133.8 ± 13.2	131.3 ± 13.6	0.017
	Platelet count (×10^9^/L)	213 (178,246)	212 (181,258.25)	0.485
Sex n (%)	Male	231 (59.84)	180 (61.02)	0.757
	Female	155 (40.16)	115 (38.98)	
History of hypertension n (%)	Yes	266 (68.91)	197 (66.78)	0.555
	No	120 (31.09)	98 (33.22)	
History of diabetes mellitus, n (%)	Yes	112 (29.02)	96 (32.54)	0.322
	No	274 (70.98)	199 (67.46)	
History of stroke n (%)	Yes	93 (24.09)	126 (42.71)	<0.001
	No	293 (75.91)	169 (57.29)	
Smoking history n (%)	Yes	63 (16.32)	48 (16.27)	0.986
	No	323 (83.68)	247 (83.73)	
Alcohol drinking n (%)	Yes	40 (10.36)	41 (13.90)	0.158
	No	346 (89.64)	254 (86.10)	

Normally distributed continuous variables are expressed as mean ± standard deviation (SD) and compared using the Student's t-test. Skewed continuous variables are expressed as median (interquartile range, IQR) and compared using the Mann-Whitney U test. Categorical variables are expressed as number (percentage) and compared using the Chi-square test.

Abbreviations: SCAD, stable coronary artery disease; SBP, systolic blood pressure; DBP, diastolic blood pressure; TC, total cholesterol; TG, triglycerides; LDL-C, low-density lipoprotein cholesterol; HDL-C, high-density lipoprotein cholesterol; ALT, alanine aminotransferase; AST, aspartate aminotransferase; SCr, serum creatinine; eGFR, estimated glomerular filtration rate; BUN, blood urea nitrogen; UA, uric acid; FT3, free triiodothyronine; FT4, free thyroxine; TSH, thyroid-stimulating hormone.

### 4.6 Multivariate analysis of risk factors and predictive model construction

To further identify independent risk factors for frequent hospitalization, variables that achieved statistical significance in the univariate analysis were incorporated into a multivariable logistic regression model (Table 5). The analysis revealed that, after adjusting for potential confounders, a history of stroke (OR = 2.108, 95% CI: 1.509–2.946, *P* < 0.001) remained the sole independent risk factor for frequent hospitalization. Conversely, age, DBP, FT3, and hemoglobin did not retain their independent predictive value in this adjusted model (all *P* > 0.05).

**TABLE 5:** Multivariable logistic regression analysis of risk factors associated with frequent hospitalization.

Variables		B	SE	Wald χ^2^	*P* value	OR	OR 95% CI
Age		0.015	0.008	3.432	0.064	1.016	0.999–1.032
History of stroke	No	—	—	—	—	1	—
	Yes	0.746	0.171	19.109	<0.001	2.108	1.509–2.946
DBP		−0.0072	0.0060	1.416	0.234	0.993	0.981–1.005
Hemoglobin		−0.006	0.006	1.014	0.314	0.994	0.982–1.006
FT3		−0.113	0.092	1.517	0.218	0.893	0.746–1.069

Dependent variable: frequent hospitalization (Yes = 1, No = 0).

Abbreviations: B, unstandardized regression coefficient; SE, standard error; OR, odds ratio; CI, confidence interval; DBP, diastolic blood pressure; FT3, free triiodothyronine.

### 4.7 Construction, performance evaluation, and internal validation of the predictive model

**FIGURE 1:**
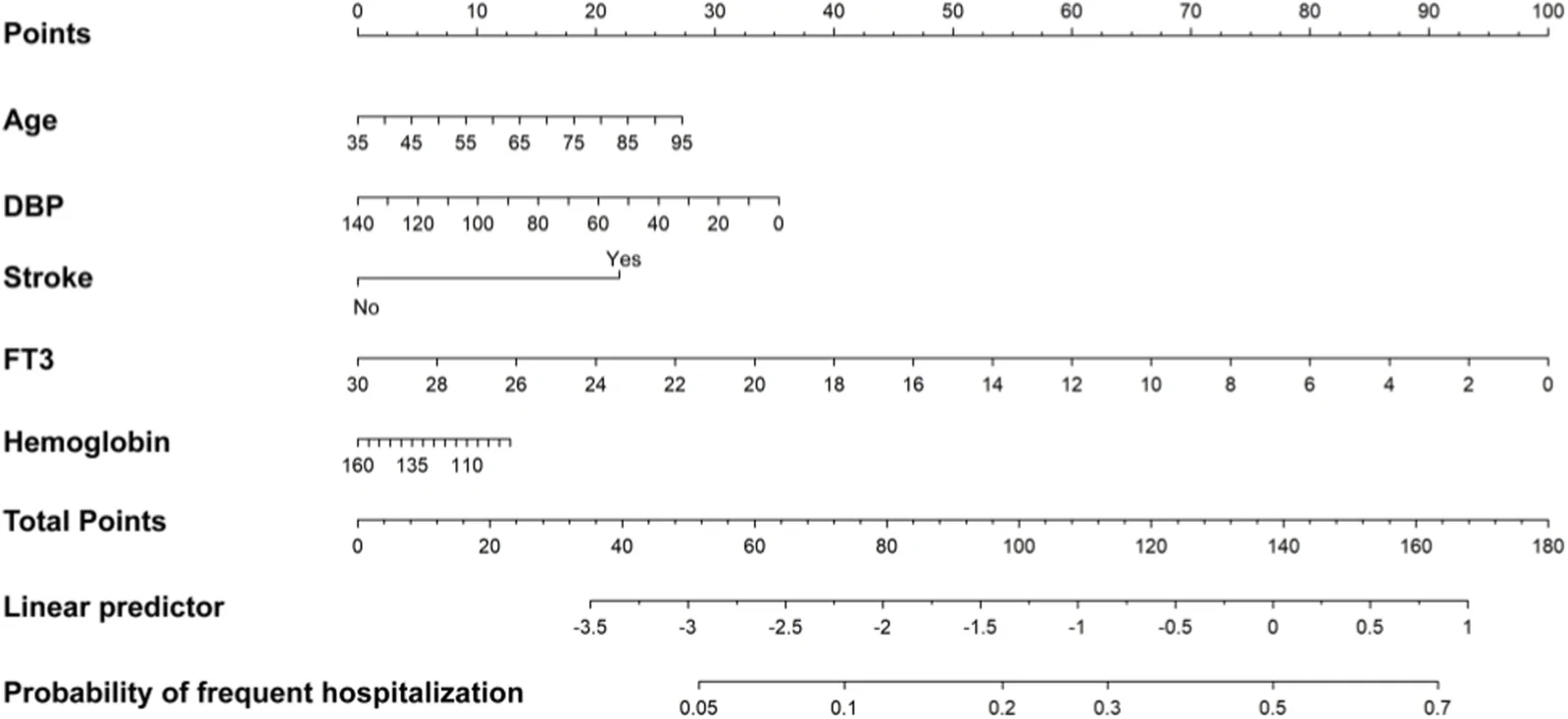
Nomogram for predicting the risk of frequent hospitalization. Notes: To use the nomogram, locate the patient’s value on the corresponding variable axis and draw a vertical line straight upward to the “Points” axis to determine the score for each variable. Sum the scores of all five variables and locate this sum on the “Total Points” axis. Finally, draw a vertical line straight downward to the bottom “Risk” axis to obtain the patient’s predicted probability of frequent hospitalization.

**FIGURE 2:**
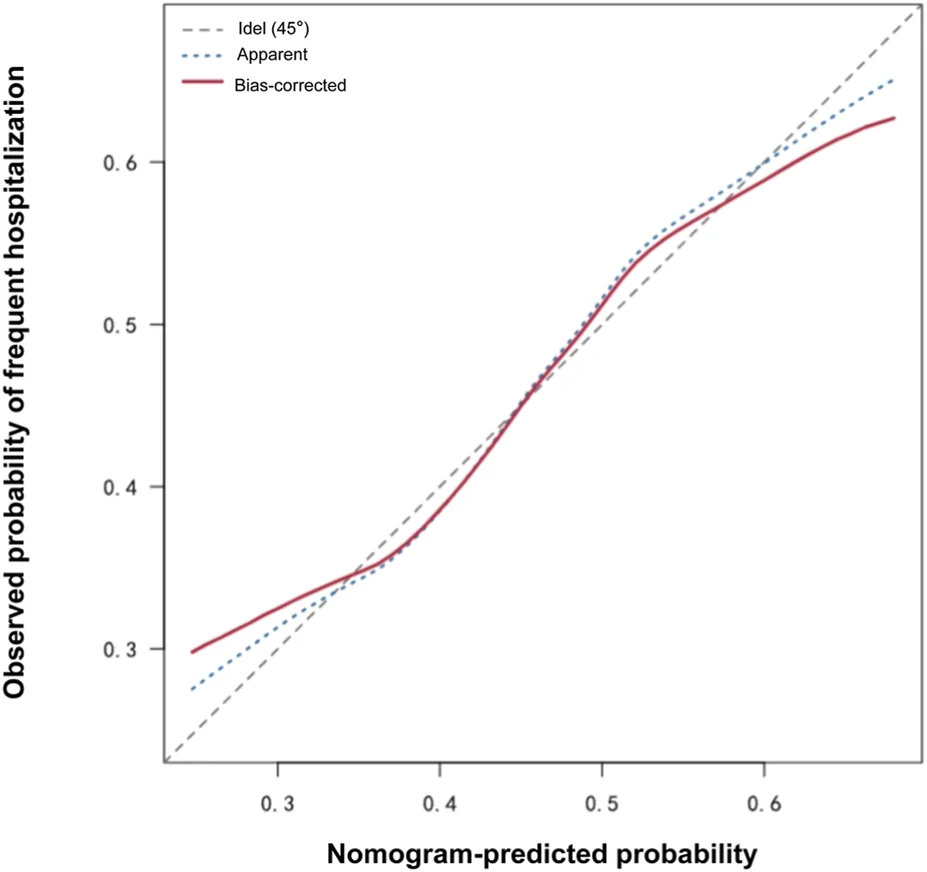
Calibration curve of the nomogram. Notes: The x-axis represents the nomogram-predicted probability of frequent hospitalization, and the y-axis represents the actual observed probability. The diagonal dashed line serves as the ideal reference line, indicating perfect agreement between the predicted probabilities and actual outcomes. Both the apparent curve and the bias-corrected curve closely align with the ideal reference line. Furthermore, the Hosmer-Lemeshow goodness-of-fit test yielded a non-significant result (P = 0.949), indicating excellent calibration and predictive agreement of the model.

Guided by the multivariable regression analysis alongside clinical relevance, a nomogram was constructed to predict the risk of frequent hospitalization by integrating five variables, including age, DBP, a history of stroke, FT3, and hemoglobin (Figure 1). Receiver operating characteristic (ROC) curve analysis (Figure 2) yielded an area under the curve (AUC) of 0.632 (95% CI: 0.590–0.674), demonstrating moderate discriminative ability for the model.

**FIGURE 3:**
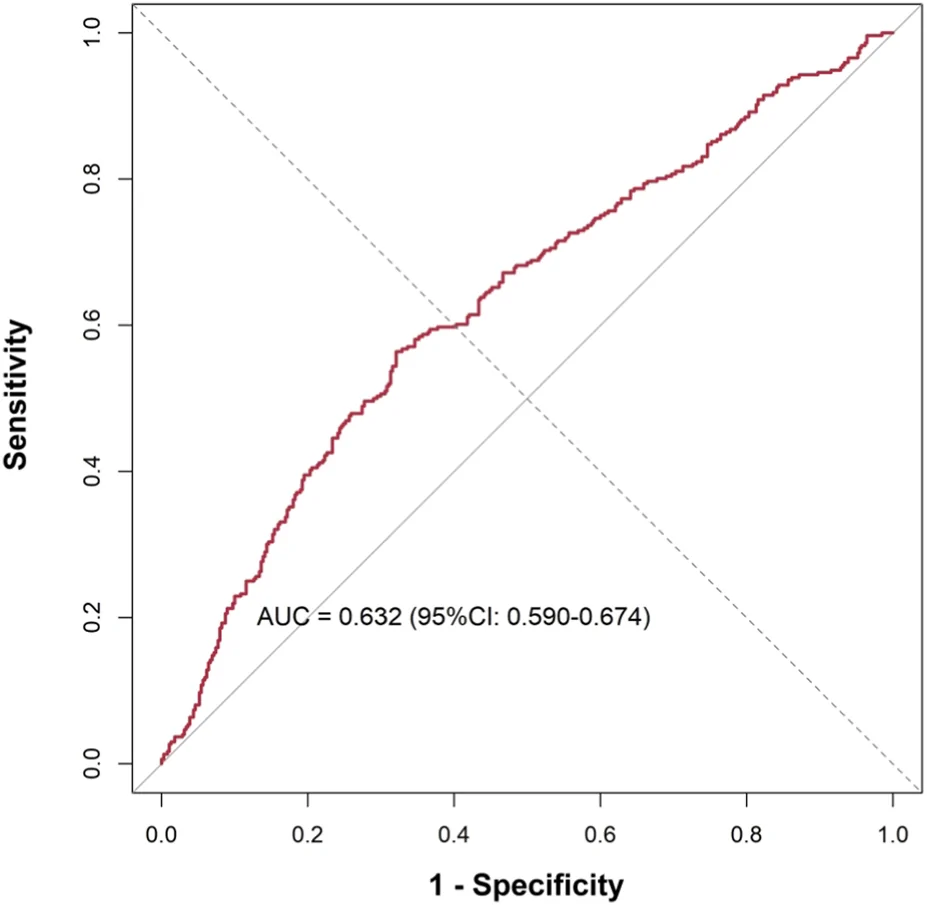
Receiver operating characteristic (ROC) curve of the nomogram. Notes: The solid red line represents the ROC curve of the nomogram for predicting the risk of frequent hospitalization. The diagonal dashed gray line represents the reference line of no discrimination (AUC = 0.500). The area under the curve (AUC/C-statistic) of the model is 0.632 (95% CI: 0.590–0.674), indicating that the prediction model possesses moderate discrimination.

Regarding model calibration, the Hosmer-Lemeshow goodness-of-fit test yielded a P value of 0.949, indicating no significant deviation between the predicted probabilities and the actual observed outcomes. This was visually corroborated by the calibration plot (Figure 3), which illustrated that both the apparent and bias-corrected curves aligned closely with the ideal 45-degree reference line. Collectively, these results confirm that the nomogram possesses excellent calibration and predictive consistency.

### 4.8 Clinical symptom characteristics and co-occurrence network analysis

**FIGURE 4:**
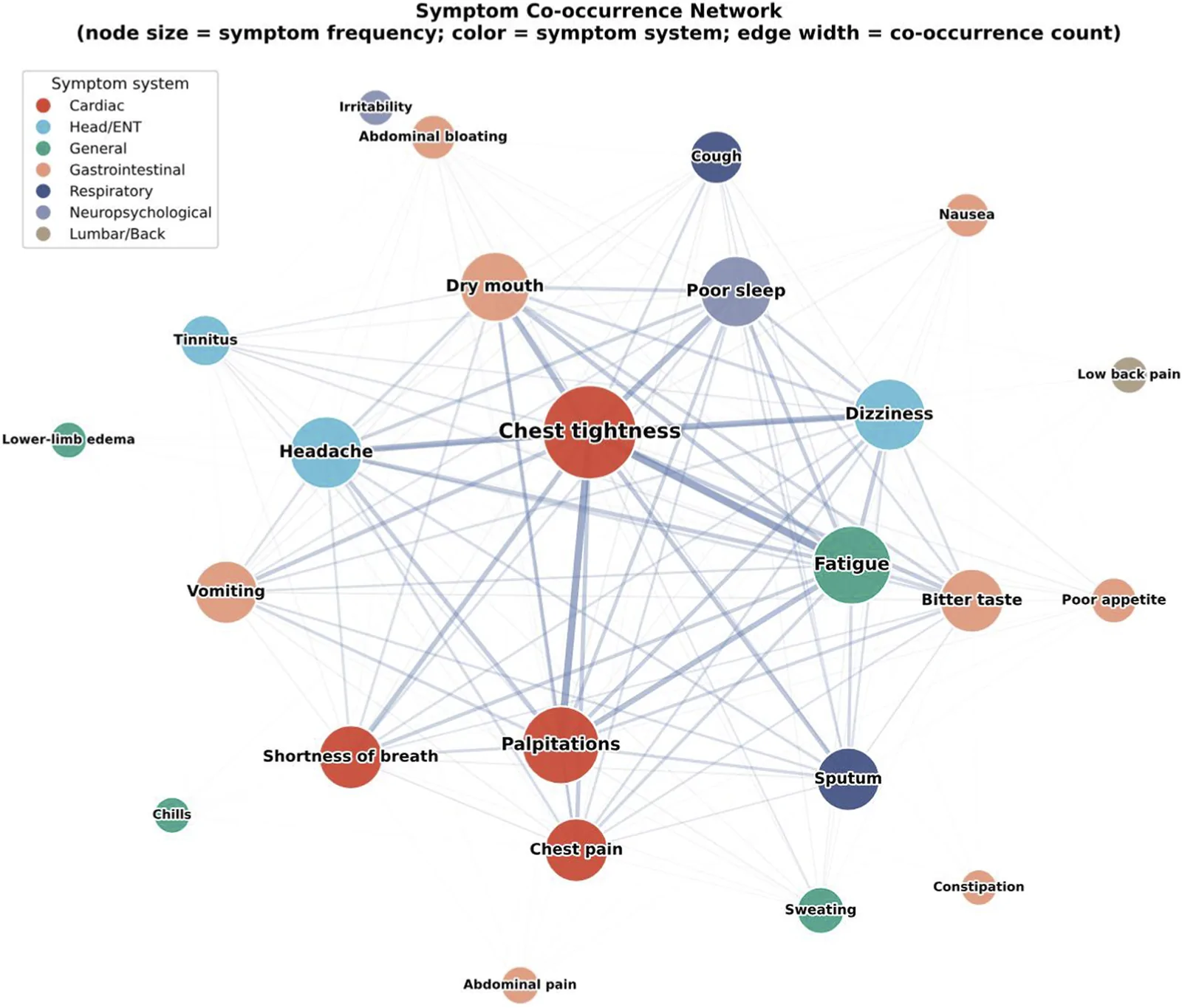
Co-occurrence network of high-frequency clinical symptoms. Notes: Circular nodes represent distinct clinical symptoms. The size of each node is proportional to the occurrence frequency of the symptom (i.e., larger nodes indicate higher frequencies). Node colors denote different symptom system categories. The edges (lines) connecting the nodes indicate symptom co-occurrence, with the thickness of the edges being proportional to the co-occurrence frequency. Symptoms positioned at the center of the network are identified as core symptoms.

Among the 686 patients, the most prevalent core clinical symptoms were chest tightness (580 cases, 84.5%), fatigue (384 cases, 56.0%), and palpitations (371 cases, 54.1%). Regarding system distribution, cardiac symptoms (such as chest tightness, palpitations, and shortness of breath) and systemic symptoms (such as fatigue) were predominant (Table 6). The symptom co-occurrence matrix analysis revealed that the top three frequent symptom pairs were chest tightness and fatigue (330 co-occurrences), chest tightness and palpitations (315 co-occurrences), alongside chest tightness and dizziness (265 co-occurrences). As illustrated in Figure 4, chest tightness, fatigue, and palpitations constituted the core hub nodes of the symptom network, forming extensive interconnections with peripheral symptoms such as nausea, constipation, and low back pain.

**TABLE 6:** Top 10 individual symptoms and frequent co-occurring symptom combinations.

Rank	Symptom	System category	Frequency (%)	Rank	Co-occurring symptoms	Count	Combination type
1	Chest tightness	Cardiovascular	580 (84.5)	1	Chest tightness – Fatigue	330	Cross-system
2	Fatigue	Systemic	384 (56.0)	2	Chest tightness – Palpitations	315	Intra-system
3	Palpitations	Cardiovascular	371 (54.1)	3	Chest tightness – Dizziness	265	Cross-system
4	Headache	Neurological	313 (45.6)	4	Chest tightness – Headache	261	Cross-system
5	Dizziness	Neurological	308 (44.9)	5	Chest tightness – Poor sleep	252	Cross-system
6	Poor sleep	Neuropsychiatric	300 (43.7)	6	Chest tightness – Dry mouth	251	Cross-system
7	Dry mouth	Gastrointestinal	285 (41.5)	7	Palpitations – Fatigue	230	Cross-system
8	Shortness of breath	Cardiovascular	223 (32.5)	8	Chest tightness – Shortness of breath	208	Intra-system
9	Bitter taste	Gastrointestinal	222 (32.4)	9	Chest tightness – Expectoration	194	Cross-system
10	Chest pain	Cardiovascular	221 (32.2)	10	Fatigue – Dry mouth	192	Cross-system

Intra-system: Co-occurring symptoms belonging to the same physiological system category. Cross-system: Co-occurring symptoms belonging to different physiological system categories.

Further analysis of the system categories involved in these associations revealed that, out of 11,315 total co-occurrences, inter-system symptom combinations accounted for a substantial 84.8% (log-transformed proportion of 83.8%). This proportion was considerably higher than that of intra-system co-occurrences (15.2%). These findings indicate that the clinical manifestations in this patient population do not represent isolated impairments of a single system. Rather, they reflect a complex pathological state characterized by the interaction of multiple systems, including the cardiac, systemic, neurological, and gastrointestinal systems.

## 5 Discussion

In this real-world, IPTW-adjusted cohort study, we demonstrated that adjunctive therapy with the ASM botanical drug pair significantly reduced LDL-C levels and improved systemic metabolic homeostasis in patients with SCAD. Furthermore, we successfully developed a clinical prognostic nomogram for frequent readmissions and mapped the core symptom network of SCAD, fully illustrating the multi-target and holistic regulatory characteristics of this TCM intervention.

In TCM theory, Qi deficiency and blood stasis constitute the fundamental pathogenesis of SCAD, which closely parallels the modern pathological cascade of impaired hemodynamics and subsequent lipid-driven atherosclerosis. The combination of Astragalus (replenishing Qi) and Salvia (invigorating blood) addresses both the root causes and manifestations of the disease (Ni et al., 2024). Crucially, by utilizing objective clinical endpoints, specifically the significant reduction in LDL-C and the elevation of vasoprotective HDL-C, our study provides quantifiable, modern evidence supporting this traditional therapeutic rationale. These objective lipid improvements align perfectly with modern pharmacological mechanisms, as lipid dysregulation is the initiating factor in atherogenesis (Zaina et al., 2005). Tanshinone IIA, the core active metabolite of Salvia, regulates lipid metabolism by inhibiting cholesterol synthesis and enhancing LDL clearance (Hu et al., 2017; Feng et al., 2024; Chen et al., 2016). Concurrently, Astragaloside IV effectively reduces serum ox-LDL levels and mitigates lipid deposition by promoting cholesterol efflux (Li T. et al., 2023). Together, these metabolites exert synergistic effects in stabilizing plaques and improving endothelial function, serving as a powerful adjunct to conventional therapies.

Beyond lipid profiles, the ASM drug pair modulated systemic indicators, including heart rate, monocytes, and thyroid hormones (FT3 and FT4). Interestingly, a physiological paradox was observed. Although FT3 and FT4 concentrations were statistically higher in the ASM group, which typically activates cardiac β-adrenergic receptors and elevates heart rate, the patients’ heart rates remained stable or even slightly decreased. This can be attributed to two factors. First, the FT3 and FT4 elevations remained firmly within the normal physiological range, reflecting a mild modulation of basal energy metabolism rather than pathological hyperthyroidism. Second, the unique sympatholytic properties of *Salvia miltiorrhiza* counteract excessive sympathetic excitation (Buenafe et al., 2013), effectively neutralizing potential tachycardic effects and sustaining cardiac electrophysiological stability. Taken together with the comparable liver and kidney function profiles and routine blood parameters between the two groups, we found no evidence that this preparation increased the risk of systemic toxicity or bleeding. This further corroborates the clinical safety of incorporating the ASM botanical drug pair into conventional therapy. This safety assessment, based on objective laboratory data, is highly consistent with the results of previous clinical studies on similar formulations (Zhang, 2024). It also implies that the drug pair plays a distinct systemic regulatory role in maintaining the internal environment and basal metabolic homeostasis of patients.

Regarding long-term prognosis, multivariate regression identified a history of stroke as the sole independent risk factor for frequent hospitalizations (≥3 times). This is clinically sound, as SCAD patients with concomitant stroke generally exhibit more extensive systemic atherosclerosis and a fragile microcirculatory compensatory capacity, rendering cardio-cerebral comorbidity highly challenging to manage. By incorporating parameters such as age and blood pressure, we constructed a prognostic nomogram. Although its area under the curve (AUC = 0.632) indicated moderate discriminative ability, the model demonstrated excellent calibration (*P* = 0.949). Similar to other recently validated cardiovascular nomograms (Li N. et al., 2023; Han et al., 2025; Xiao et al., 2021), our tool effectively translates complex regression equations into an intuitive visual format. This enables clinicians to rapidly stratify readmission risks and implement more proactive, individualized follow-up strategies.

Finally, through in-depth data mining of the patients’ clinical symptom networks using the Apriori algorithm, this study revealed that chest tightness serves as the central hub of the entire symptom network, forming high-frequency symptom clusters with dizziness, palpitations, and fatigue. Interestingly, these topological features highly align with the therapeutic rationale of the ASM botanical drug pair. Specifically, this botanical combination acts by replenishing Qi to ameliorate fatigue and dizziness, while invigorating blood circulation to relieve chest tightness and palpitations. Consequently, this targeted intervention substantially enhances the overall quality of life for patients at the symptomatic level.

### 5.1 Limitations

Despite employing an IPTW design and multidimensional statistical analyses to minimize bias, this study possesses several inherent limitations. First, due to its retrospective observational nature, certain unmeasured confounders, such as dietary habits and lifestyle, could not be fully adjusted. Moreover, while we mapped the clinical symptom network, these symptoms were evaluated based on electronic medical records (EMRs) rather than standardized prospective quantitative scales, introducing potential subjectivity. To mitigate this inherent subjectivity, our study deliberately anchored its core efficacy evaluation on objective and quantifiable clinical endpoints, specifically longitudinal LDL-C trajectories and verifiable hospital readmission rates, thereby ensuring the scientific rigor of our conclusions.

Second, extracting longitudinal EMR data over a 10-year follow-up precluded the dynamic capture of precise frequencies, dosage adjustments, and long-term adherence to concurrent standard cardiovascular medications, such as statins and beta-blockers. Although IPTW robustly balanced baseline profiles, dynamic variations in longitudinal lipid-lowering therapies may still act as unmeasured confounding variables. Third, in real-world settings, Astragalus and Salvia are frequently prescribed alongside other botanical medicines. While we successfully isolated the exposure effects of this core pair, potential interactive effects from concomitant botanical drugs and the exact dose-response relationships remain challenging to entirely disentangle.

Fourth, owing to the nature of retrospective real-world clinical data, our study could not obtain detailed extraction methods or batch-specific phytochemical profiles for the botanical interventions. EMRs inherently only document general dosage forms (such as decoctions, granules, or Chinese patent medicines) and prescribed weights. Nevertheless, this limitation is substantially mitigated because all botanical medicines dispensed in the study institution are legally mandated to strictly comply with the monographs of the Chinese Pharmacopoeia (ChP 2020, as detailed in Table 1), ensuring a standardized baseline quality. Fifth, the predictive nomogram developed herein was internally validated using bootstrapping techniques; however, it lacks external validation across independent clinical cohorts.

Finally, the single-center design limits the generalizability of our findings to broader populations. Consequently, future multi-center, prospective, and large-scale randomized controlled trials are warranted. These future studies should ideally incorporate hard cardiovascular endpoints (MACE) and advanced technologies, such as untargeted metabolomics, to further validate the long-term clinical efficacy and elucidate the underlying molecular mechanisms of the ASM botanical drug pair.

## 6 Conclusion

Based on IPTW-adjusted real-world clinical data, this study demonstrates that adjunctive therapy with the ASM botanical drug pair effectively reduces LDL-C levels and promotes physiological homeostasis in patients with SCAD. Importantly, this treatment also exhibits a favorable long-term safety profile. Furthermore, we identified a history of stroke as an independent risk factor for frequent hospitalizations in this population. The clinical nomogram developed herein can reliably stratify these high-risk individuals. Beyond improving objective biochemical indices, our findings show that the ASM pair significantly alleviates core somatic symptoms, particularly chest tightness. This indicates a comprehensive clinical benefit for patient care. Ultimately, this study provides solid real-world evidence to optimize the long-term management of SCAD, and supports the broader application of integrative therapies in clinical practice.

## Data Availability

The data analyzed in this study is subject to the following licenses/restrictions: Please contact the first author if required. Requests to access these datasets should be directed to ff9898213@163.com.
